# Changes in circulating immune cells and cytokines after high-intensity aerobic exercise in patients with prostate cancer undergoing active surveillance from the ERASE randomized controlled trial

**DOI:** 10.3389/fimmu.2026.1923463

**Published:** 2026-09-07

**Authors:** Dong-Woo Kang, Dhruvesh Patel, Catherine J. Field, Normand G. Boulé, Adrian S. Fairey, Keri L. Schadler, Kerry S. Courneya

**Affiliations:** 1 Public Health Sciences Division, Fred Hutchinson Cancer Center, Seattle, WA, United States; 2 Department of Epidemiology, School of Public Health, University of Washington, Seattle, WA, United States; 3 Department of Agricultural, Food and Nutritional Science, Faculty of Agricultural, Life and Environmental Sciences, University of Alberta, Edmonton, AB, Canada; 4 Faculty of Kinesiology, Sport, and Recreation, College of Health Sciences, University of Alberta, Edmonton, AB, Canada; 5 Division of Urology, Department of Surgery, Faculty of Medicine & Dentistry, University of Alberta, Edmonton, AB, Canada; 6 Department of Pediatrics, University of Texas MD Anderson Cancer Center, Houston, TX, United States

**Keywords:** active surveillance, prostate cancer, exercise, high-intensity interval training, immune function, natural killer cells, cytokines, randomized controlled trial

## Abstract

**Background:**

Exercise can modulate immune function and inflammatory signaling in cancer populations; however, its effects on the immune system in men with localized prostate cancer undergoing active surveillance are unknown. This study examined the effects of high-intensity interval training (HIIT) on circulating immune cell phenotypes, function, and systemic cytokine levels in this clinical setting.

**Methods:**

This was a secondary analysis of the Exercise During Active Surveillance for Prostate Cancer (ERASE) trial, a single-center randomized controlled trial. Fifty-two men with localized prostate cancer on active surveillance were randomized to a 12-week supervised aerobic HIIT program (n=26) or usual care (n=26). HIIT consisted of three sessions per week at 85% to 95% of peak oxygen consumption. Fasting blood samples were collected at baseline and post-intervention. Changes in immune cell subsets (T cells, B cells, and NK cells; flow cytometry), NK cell cytotoxicity, and plasma cytokine concentrations were assessed. Analyses of covariance were used to compare between-group differences.

**Results:**

The HIIT group attended 96% of prescribed sessions. Compared with usual care, the HIIT group showed a significant increase in the proportion of the circulating major NK-cell subset (CD3−CD56+CD16+) (adjusted between-group difference, 2.0%; 95% CI, 0.3 to 3.7; p=0.024) and PBMC-mediated cytotoxicity against K562 (2.9%; 95% CI, 0.3 to 5.4; p=0.031). A significant between-group decrease was also observed in basophil counts (-0.04 ×10^9^/L; 95% CI, -0.07 to -0.01; *p* = 0.011), with no differences in other leukocyte populations. HIIT significantly reduced plasma levels of IL-4 (-0.02 pg/mL; 95% CI, -0.03 to -0.01; *p* = 0.011) and IL-12p70 (-0.14 pg/mL; 95% CI, -0.23 to -0.06; *p* = 0.002) compared with usual care. No significant between-group differences were observed in T cell or B cell subsets, or other inflammatory cytokines including IL-6 and TNF-α.

**Conclusions:**

A 12-week supervised HIIT program significantly increased the proportion of the circulating major NK-cell subset and PBMC-mediated cytotoxicity while reducing IL-4 and IL-12p70 concentrations in men with prostate cancer undergoing active surveillance. Because these were peripheral-blood measures rather than tumor-level readouts, and given the exploratory design and number of comparisons, these findings should be interpreted as hypothesis-generating and require confirmation in larger, adequately powered studies.

## Introduction

The immune system plays a central role in cancer surveillance, progression, and therapeutic responsiveness. Both innate and adaptive immune cells, such as natural killer (NK) cells, cytotoxic T cells, and myeloid-derived suppressor cells, contribute to the host’s ability to detect and eliminate malignant cells (1). In parallel, cytokines and chemokines regulate immune cell trafficking, activation, and effector function within the tumor microenvironment and systemically (2, 3). In prostate cancer specifically, studies have implicated systemic inflammation, immunosenescence, and cytokine dysregulation in disease progression and poor clinical outcomes (4). Interventions that can modulate immune function and inflammatory signaling are therefore of interest in the context of early-stage prostate cancer, particularly for patients undergoing active surveillance.

Emerging preclinical and clinical evidence suggest that exercise can elicit significant alterations in immune cell mobilization, phenotype, and function in both healthy and clinical populations (5). Acute bouts of aerobic exercise transiently elevate circulating levels of NK cells, effector T cells, and cytokines such as IL-6 and TNF-α. Although IL-6 is often classified as pro-inflammatory, exercise-induced IL-6 is released predominantly by contracting skeletal muscle as a myokine and exerts largely anti-inflammatory and metabolic effects, in contrast to the chronic, trans-signaling-mediated IL-6 associated with low-grade inflammation (6, 7). With regular training, these acute responses are accompanied by enhanced immunosurveillance and reduced chronic inflammation (6). In cancer populations, exercise has shown potential to modulate immune parameters, including increased tumor-infiltrating lymphocytes and improved NK cell cytotoxicity, which may in turn suppress cancer progression (8, 9). However, the vast majority of current evidence is derived from animal models, and little is known about immune responses to exercise in the context of clinical populations with extant cancer, such as active surveillance (10).

The Exercise During Active Surveillance for Prostate Cancer (ERASE) trial was a randomized controlled trial designed to evaluate the effects of a 12-week supervised high-intensity interval training (HIIT) intervention on cardiorespiratory fitness and prostate cancer progression biomarkers in men with localized prostate cancer under active surveillance (10, 11). In the primary paper, we reported that HIIT significantly reduced PSA levels, PSA velocity, and LNCaP cell growth (11). In this paper, we report pre-specified secondary outcomes focused on circulating immune cells and cytokines. Specifically, we examined how HIIT influenced the distribution of key immune cell subsets and systemic cytokine levels and hypothesized that high-intensity aerobic training may positively modulate cytokines and immune pathway in this clinical population.

## Methods

### Study design and participants

The ERASE trial was a single-center, two-arm, randomized controlled trial conducted at the University of Alberta and Cross Cancer Institute, Edmonton, AB, Canada (NCT03203460) (10). The trial enrolled 52 men diagnosed with localized prostate cancer under active surveillance who were not currently engaging in vigorous-intensity physical activity (11). Eligibility criteria included: age ≥18 years, histologically confirmed very low to favorable intermediate-risk prostate cancer, and medical clearance to participate in high-intensity exercise and cardiopulmonary testing. Exclusion criteria included plans for curative treatment during the study period and contraindications to vigorous physical activity. Following baseline testing, participants were randomized in a 1:1 ratio using block randomization to either a 12-week HIIT intervention or a usual care (UC) group. Allocation was concealed using a centralized computer-based system, and outcome assessors for immune endpoints were blinded to group assignments. Details of the study design have been reported elsewhere (10, 11).

### Interventions

Participants randomized to the exercise group completed a 12-week, supervised HIIT program, performed three times per week at a university-based fitness facility located adjacent to the cancer center. All sessions were conducted on a treadmill and were individually prescribed based on each participant’s baseline peak oxygen consumption (VO_2_peak) obtained from a graded cardiopulmonary exercise test. Each session began with a 5-minute warm-up at 60% of VO_2_peak, followed by alternating 2-minute bouts of high-intensity exercise at 85% to 95% of VO_2_peak and 2-minute active recovery periods at 40% of VO_2_peak. Sessions concluded with a 5-minute cooldown at 30% of VO_2_peak. The number of intervals progressed from 5 during the first week to 8 by week 5, resulting in total session durations increasing from 28 to 40 minutes. Although VO_2_ was not directly measured during each exercise bout, treadmill speed and grade were individually calibrated from baseline testing to match target intensities. All sessions were supervised by study personnel trained in clinical exercise physiology. Participants were asked to refrain from any additional structured or vigorous-intensity physical activity during the study period. Adherence was defined as attending at least 80% of the scheduled exercise sessions, and all sessions were monitored for exercise fidelity, including recording of heart rate and perceived exertion. Participants randomized to the UC group were instructed to continue their habitual lifestyle and not initiate any new structured exercise or high-intensity physical activity during the 12-week intervention period. To support retention and mitigate group differences in access to exercise post-trial, participants in the UC group were offered an opportunity to engage in a 4-week supervised HIIT program at the same facility used by the intervention group, or a referral to a 12-week community-based exercise program tailored for individuals with cancer. Both post-trial exercise options were presented after completion of the final assessments.

### Blood collection and processing

Venous blood samples were collected after 12 hours of fasting at the Kaye Edmonton Clinic Laboratory Services and processed in the Li Ka Shing Centre for Health Research Innovation at the University of Alberta. To minimize the influence of the acute exercise response, post-intervention blood samples were collected at least 72 hours after the final HIIT session, capturing chronic training adaptations rather than transient post-exercise changes. Per the trial protocol, participants in both the HIIT and usual-care groups were instructed to avoid structured or vigorous physical activity for approximately 72 hours before every blood draw, so post-intervention samples in both groups were collected under equivalent resting conditions. Whole blood was drawn into coated EDTA tubes and processed within one hour. One set of whole blood tubes were assessed for Complete Blood Count (CBC) and differential at the Royal Alexandra Hospital - Laboratory Services (Dyna Lab/Alberta Precision Laboratories) using an automated hematology analyzer. The second set of whole blood tubes were centrifuged at 2860 rpm (750x *g)* for 10 minutes to obtain plasma, which was frozen and banked at -80°C for cytokines and hormones analysis. The remaining sample was diluted to 6 mL with phosphate-buffered saline (PBS) containing 10 g/L bovine serum albumin (Sigma-Aldrich). Peripheral blood mononuclear cells (PBMC) were separated by density gradient of Histopaque-1077 (Sigma-Aldrich) as previously described (12). Briefly, 4 mL diluted blood solution was layered on top of 4 mL of Histopaque 1077 and centrifuged at 1800 rpm (700x g) at 21°C for 30 minutes with no brake. The white blood cell (WBC) band was recovered from the gradient interface and cells were resuspended in complete cell culture media (CCM). Cells were counted on a hemacytometer and trypan blue dye exclusion. Fresh cells were diluted to 1x10^6^ cells/mL and used for PBMC phenotype analyses. Remaining cells were suspended in CCM containing dimethyl sulfoxide (DMSO) and transferred to sterile cryovial, followed by slow freeze in Mr. Frosty Freezing Container (ThermoFisher Scientific) and stored in liquid nitrogen.

### Plasma cytokine analysis

Plasma cytokine levels were analyzed using commercially available electrochemiluminescence kits (pro-inflammatory panel-1, Meso Scale Discover). Cytokine detection ranges were as follows: interferon-γ (IFN-γ, 0.37–938 pg/mL), interleukin-2 (IL-2, 0.09–938 pg/mL), IL-4 (0.02–158 pg/mL), IL-6 (0.06–488 pg/mL), IL-8 (0.07–375 pg/mL), IL-10 (0.04–233 pg/mL), IL-12p70 (0.11–315 pg/mL), IL-13 (0.24–353 pg/mL), and tumor necrosis factor-α (TNF-α, 0.04–248 pg/mL), and high-sensitivity C-reactive protein (hs-CRP, <0.8 mg/L). All samples were measured in duplicate with a coefficient of variation (CV) less than 15%, and the concentrations were determined based on the standard curves.

### Cell phenotyping assay

The phenotype of PBMC was assessed by direct immunofluorescence assay as previously described (13, 14). Briefly, the isolated PBMCs were incubated for 30 minutes with the following fluorophore-conjugated monoclonal antibodies (mAbs; all from Biolegend), listed as target marker–fluorochrome (clone): CD45RA-PE (HI100), CD45RO-PerCP (UCHL1), CD4-APC (OKT4), CD107a-PE (H4A3), CD56-APC (5.1H11), CD16-PerCP (3G8), CD25-PE (BC96), CD11c-FITC (3.9), CD54-PE (HA58), CD103-PerCP (Ber-ACT8), and HLA-DR-APC (LN3); the remaining lineage and exclusion markers (CD3, CD8, CD19, CD27, CD70, and IgM) were pre-conjugated mouse anti-human mAbs used according to the manufacturer’s staining recommendations; clone and catalog identifiers for these reagents are available from the corresponding author on request. The cells were then washed and fixed in 1% paraformaldehyde (1% wt/wt) in PBS. All samples were acquired within 24 hours by flow cytometry (BD LSRFortessa, Becton Dickinson) and analyzed according to the relative fluorescence intensity using FlowJo v10 (BD Biosciences).

Compensation controls, isotype controls, and unstained controls were used to ensure data quality and to guide gating, and antibody/fluorochrome combinations were selected after troubleshooting to minimize spectral overlap. Fluorescence-minus-one (FMO) controls were not used because spectral overlap among the selected fluorochromes was negligible, giving clear separation between positive and negative populations. Doublets were excluded by gating single cells on forward-scatter area versus forward-scatter height. A live/dead viability dye was not included; instead, cell viability was assessed by hemacytometer with trypan blue dye exclusion, cells were resuspended at 1×10^6^ cells/mL, staining was performed on freshly collected blood (minimizing dead cells), and the forward-scatter threshold was set to exclude debris and dead cells.

The gating hierarchy was as follows. After excluding debris and doublets, lymphocytes were gated on forward- and side-scatter. T cells were identified as CD3+, with helper (CD3+CD4+) and cytotoxic (CD3+CD8+) subsets; activated cells were defined identically in both compartments as CD25+ (CD25/IL-2Rα+), and CD45RA+ (predominantly naïve) and CD45RO+ (memory) subsets were defined within CD3+CD4+. B cells were identified as CD19+, with CD11c+CD19+ (atypical/age-associated memory) and IgM+CD19+ subsets. The major NK-cell subset was defined as CD3−CD56+CD16+ with CD3− exclusion applied; a CD27+CD16+CD56+ NK-associated subset was assessed in a separate tube that did not include a CD3 channel, so CD3−/NKT exclusion and CD11b-based maturation staging were not possible for that subset.

### NK cell cytotoxicity assay

Natural Killer cell cytotoxicity was analyzed using cryopreserved PBMC using the CyQUANT Lactate dehydrogenase (LDH) Cytotoxicity Assay kit. Cells were thawed in water bath at 37°C, washed and resuspended in CCM, and incubated overnight at 37°C with 5% CO2. The effector-to-target (E:T) ratio was 5:1 with a 4-hour incubation, determined during protocol development. Cell viability was assessed by hemocytometer with trypan blue dye exclusion before setting up the assay; per-sample post-thaw viability was not recorded, but was approximately 70% in protocol-development and troubleshooting experiments. Effector PBMCs were cocultured with target K562 cells for 4 hours at 37°C with 5% CO2. All patient samples, including paired pre- and post-intervention samples from both arms, were thawed and analyzed on the same day. Appropriate controls to measure maximum LDH activity, spontaneous LDH activity was also prepared as per the kit protocol. All the samples and controls were measured in triplicate and read at 490 nm and 680 nm on spectrophotometer Sinergy H1 (Agilent BioTek). LDH activity was calculated by subtracting the 680 nm absorbance value (background) from the 490 nm absorbance value. The percent (%) cytotoxicity was calculated using the formula: (sample LDH activity - spontaneous LDH activity)/(Maximum LDH activity - spontaneous LDH activity) x 100.

### Statistical analysis

Analyses followed the intention-to-treat principle. Continuous immune markers were assessed for distributional assumptions, and skewed variables were log-transformed to improve normality and variance stability (transformations are noted in the result tables). For these variables, all subsequent analyses, including outlier screening, were performed on the log-transformed scale. Missing cases were excluded listwise within each model, and outliers were identified using studentized residuals (± 3.0) from the corresponding model and removed from analysis. Within-group changes were evaluated using paired t-tests, and between-group differences were analyzed using analysis of covariance (ANCOVA), adjusting for baseline values and resistance exercise behavior. Moderation analyses were conducted to explore whether changes in immune markers moderated the effect of the exercise intervention on PSA levels and androgen dependent LNCaP tumor cell response. Potential moderators (IL-4, IL-12p70, NK cell %, and NK cell cytotoxicity) were selected based on significant group differences in immune responses. Linear regression models were constructed including group assignment, the changes in immune markers, and their interaction terms. The dependent variables were PSA change and LNCaP tumor cell viability changes. For each model, we report the unstandardized coefficient and 95% confidence interval for the interaction term, standardized beta (β) for effect size, and R² for model fit. In the ERASE trial, all immune-cell and cytokine measures were pre-specified secondary/exploratory outcomes (NCT03203460) (10). Given the large number of comparisons across the CBC differential, PBMC subsets, and cytokines and the exploratory design, we base interpretation on the magnitude and precision of the effect sizes rather than on statistical significance alone. Consistent with this exploratory, hypothesis-generating design, we did not adjust p values for multiple comparisons, and unadjusted p values are reported throughout. For log-transformed outcomes, within- and between-group inferences were drawn on the log scale, as indicated in the table footnotes. All immune findings are framed as hypothesis-generating and requiring independent replication. Statistical significance was set at p < 0.05. All analyses were conducted using SPSS version 26 (IBM Corp., Armonk, NY).

## Results

The participant flow and their baseline demographic and medical profiles are shown in **Table 1**. In brief, the mean (SD) age was 63.4 (7.1) years, 89% self-identified as White, and most presented with T1c, Gleason grade 6 (3 + 3) disease. Participants in the HIIT group attended 96% of prescribed sessions with full adherence to the prescribed intensity and duration. The number of participants contributing data to each immune outcome varied by assay and is reported in the corresponding tables.

**Table 1:** Patient baseline characteristics in the ERASE trial.

Variables	Total (N = 52)	HIIT (n = 26)	Usual care (n = 26)
Age, years (Mean, SD)	63.4 (7.1)	63.9 (7.5)	62.8 (6.9)
Anthropometrics			
BMI, kg/m² (Mean, SD)	29.0 (4.7)	29.0 (5.7)	29.0 (3.5)
Waist Circumference, cm (Mean, SD)	102.3 (13.4)	101.4 (14.4)	103.3 (12.6)
Obese (BMI ≥ 30), n (%)	21 (40)	10 (38)	11 (42)
Clinical & oncologic history			
Presence of Comorbidities, n (%)	43 (83)	22 (85)	21 (81)
Arthritis or Arthralgia	31 (60)	16 (62)	15 (58)
Hypertension	16 (31)	8 (31)	8 (31)
PSA, µg/L (Mean, SD)	7.3 (3.2)	6.0 (2.3)	8.6 (3.5)
Clinical Stage T1c, n (%)	47 (90)	24 (92)	23 (89)
Gleason Score 6 (3 + 3), n (%)	50 (96)	25 (96)	25 (96)
Time on Active Surveillance, months	23.0 (25.8)	26.7 (27.0)	19.4 (24.4)
Lifestyle factors			
Smoking History (Current/Former), n (%)	30 (58)	16 (62)	14 (54)
Alcohol Consumption (Regular/Social), n (%)	45 (87)	22 (85)	23 (88)
Baseline cardiorespiratory fitness			
VO_2peak_, mL/kg/min (Mean, SD)	29.0 (6.3)	29.6 (5.8)	28.4 (6.9)

BMI, body mass index; PSA, prostate specific antigen; VO_2peak_, peak oxygen consumption.

**Figure 1:**
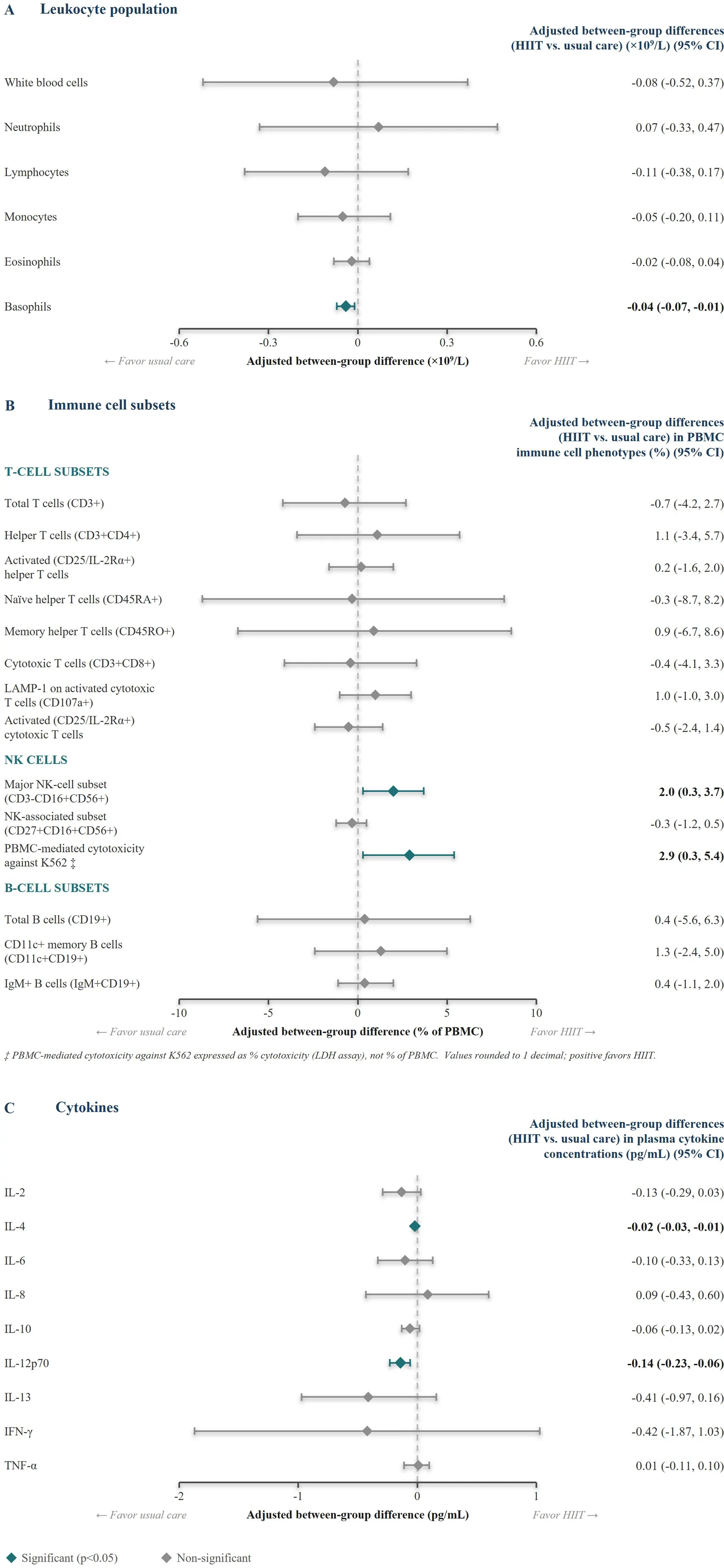
Adjusted between-group differences in circulating immune measures after 12 weeks of HIIT versus usual care (ERASE trial). Forest plots show the adjusted between-group difference (diamond) with 95% CI (horizontal line) from ANCOVA adjusting for the baseline value of each outcome and resistance-exercise behavior: **(A)** complete blood count with differential (×10^9^/L); **(B)** PBMC immune cell subsets (% of PBMC); **(C)** plasma cytokines (pg/mL). Positive values indicate higher levels with HIIT. Teal diamonds indicate statistically significant between-group differences (p<0.05); gray diamonds, non-significant. The number of participants contributing to each outcome varied by assay and is reported in **Tables 2**–**4**. PBMC-mediated cytotoxicity against K562 is expressed as % cytotoxicity (LDH assay), not % of PBMC. For log-transformed outcomes (indicated in **Tables 2**–**4**), differences are shown on the log scale; subset denominators are detailed in the **Table 3** footnote. HIIT, high-intensity interval training; CD, cluster of differentiation; LAMP-1, lysosome-associated membrane protein 1; NK, natural killer; IL, interleukin; IFN, interferon; TNF, tumor necrosis factor; CRP, C-reactive protein.

The change in CBC with differential from baseline to postintervention by randomized group are presented in **Table 2** and **Figure 1A**. There were no significant between-group differences in total white blood cells (WBC), neutrophils, lymphocytes, monocytes, or eosinophils. A statistically significant between-group difference was observed for basophils (adjusted between-group difference, -0.04 x10^9^/L; 95% CI, -0.07 to -0.01; p = 0.011); however, given the very small absolute change (0.05 to 0.03 x10^9^/L in the HIIT group), the borderline within-group change, and the multiplicity concern, this finding should be interpreted with caution and is not ascribed strong biological meaning. In within-group analyses, WBC levels significantly decreased in the HIIT group (mean change, -0.40 x10^9^/L; p = 0.029), whereas no significant change was observed in the UC group.

**Table 2:** Effects of high-intensity interval training on complete blood count with differential in men with prostate cancer under active surveillance.

Variables	Baseline	Postintervention	Within-group mean change		Adjusted between-group difference	
	Mean (SD)	Mean (SD)	Mean (95% CI)	*P*	Mean (95% CI)	*P*
White blood cell (10^9^/L)						
HIIT (n=24)	6.73 (1.60)	6.33 (1.32)	-0.40 (-0.76, -0.04)	**0.029**	-0.08 (-0.52, 0.37)	0.73
UC (n=25)	5.95 (1.13)	5.78 (1.17)	-0.17 (-0.49, 0.14)	0.27		
Neutrophils (10^9^/L)						
HIIT (n=24)	4.03 (0.99)	3.75 (1.01)	-0.28 (-0.58, 0.03)	0.073	0.07 (-0.33, 0.47)	0.73
UC (n=25)	3.43 (0.84)	3.23 (0.82)	-0.20 (-0.48, 0.07)	0.14		
Lymphocytes (10^9^/L)						
HIIT (n=24)	1.78 (0.66)	1.61 (0.57)	-0.17 (-0.40, 0.06)	0.14	-0.11 (-0.38, 0.17)	0.44
UC (n=25)	1.67 (0.58)	1.67 (0.64)	-0.02 (-0.21, 0.17)	0.83		
Monocytes (10^9^/L)						
HIIT (n=24)	0.67 (0.19)	0.67 (0.29)	0.00 (-0.09, 0.10)	0.93	-0.05 (-0.20, 0.11)	0.55
UC (n=25)	0.55 (0.16)	0.58 (0.29)	0.04 (-0.07, 0.14)	0.50		
Eosinophils (10^9^/L)						
HIIT (n=24)	0.21 (0.14)	0.25 (0.18)	0.04 (-0.01, 0.08)	0.11	-0.02 (-0.08, 0.04)	0.53
UC (n=25)	0.22 (0.11)	0.26 (0.14)	0.04 (0.00, 0.09)	0.053		
Basophils (10^9^/L)						
HIIT (n=24)	0.05 (0.05)	0.03 (0.05)	-0.02 (-0.05, 0.00)	0.096	-0.04 (-0.07, -0.01)	**0.011**
UC (n=25)	0.04 (0.05)	0.06 (0.06)	0.02 (0.00, 0.04)	**0.022**		
Neutrophil: lymphocyte ratio						
HIIT (n=23)	2.52 (0.94)	2.42 (0.93)	-0.09 (-0.42, 0.23)	0.56	0.27 (-0.10, 0.63)	0.15
UC (n=24)	2.24 (1.05)	1.98 (0.77)	-0.26 (-0.53, 0.01)	0.061		

T-test was used for within-group differences, and analysis of covariance was used for between-group differences adjusting for baseline values of the outcome and resistance exercise behavior. HIIT, high-intensity interval training; UC, usual care. Per-outcome sample sizes vary owing to assay failure, insufficient sample volume, or removal of statistical outliers identified by studentized residuals.

Bold values indicate statistical significance (p < 0.05).

We characterized PBMC from the patient’s blood samples using multi-parametric gating flow cytometry. The proportion of total T cells, helper T cells, cytotoxic T cells and total B cells in PBMC showed no significant within-group change or between-group differences (**Table 3**; **Figure 1B**). The major NK-cell subset was defined as CD3−CD56+CD16+ cells in PBMC (CD3− exclusion applied); CD16 expression distinguishes the two main NK-cell subsets, but a CD16-independent CD3−CD56+ population was not acquired. A CD27+CD16+CD56+ NK-associated subset was assessed in a separate tube that did not include a CD3 channel, so CD3−/NKT exclusion could not be applied to this subset. Compared to the UC group, the major NK-cell subset increased in the HIIT group (adjusted between-group mean change, 2.0% of PBMC; 95% CI, 0.3 to 3.7; p = 0.024). The CD27+CD16+CD56+ NK-associated subset decreased in the UC group (within-group mean change, -1.1% of PBMC; 95% CI, -2.1 to -0.1; p = 0.04). Compared to the UC group, PBMC-mediated cytotoxicity against K562 increased in the HIIT group (adjusted between-group mean change, 2.9 percentage points; 95% CI, 0.3 to 5.4; p = 0.031).

**Table 3:** Effects of high-intensity interval training on immune cells of peripheral blood mononuclear cells (PBMCs) in men with prostate cancer under active surveillance.

Variables	Baseline	Postintervention	Within-group mean change		Adjusted between-group difference	
	Mean (SD)	Mean (SD)	Mean (95% CI)	*P*	Mean (95% CI)	*P*
Total T cells (CD3+)						
HIIT (n=21)	57.3 (7.9)	57.7 (9.0)	0.5 (-2.5, 3.4)	0.75	-0.7 (-4.2, 2.7)	0.67
UC (n=24)	59.8 (10.5)	60.7 (10.4)	0.9 (-1.0, 2.8)	0.32		
Helper T cells (CD3+CD4+)						
HIIT (n=18)	75.2 (10.6)	75.4 (11.6)	0.2 (-1.6, 2.0)	0.80	1.1 (-3.4, 5.7)	0.62
UC (n=21)	63.2 (12.4)	63.7 (11.7)	0.5 (-2.8, 3.9)	0.74		
Activated (CD25/IL-2Rα+) helper T cells (CD25+ in CD3+CD4+)						
HIIT (n=18)	9.3 (3.0)	9.2 (3.3)	-0.1 (-1.6, 1.4)	0.88	0.2 (-1.6, 2.0)	0.82
UC (n=22)	9.3 (3.1)	8.8 (3.3)	-0.5 (-1.8, 0.9)	0.48		
Naïve helper T cells^*^ (CD45RA+ [predominantly naïve] in CD3+CD4+)						
HIIT (n=18)	47.5 (16.0)	47.2 (16.6)	-0.3 (-4.4, 3.8)	0.88	-0.3 (-8.7, 8.2)	0.95
UC (n=24)	37.5 (15.0)	38.6 (18.5)	1.1 (-5.2, 7.3)	0.73		
Memory helper T cells^*^ (CD45RO+ in CD3+CD4+)						
HIIT (n=19)	47.2 (17.5)	45.7 (15.9)	-1.5 (-3.6, 0.6)	0.14	0.9 (-6.7, 8.6)	0.81
UC (n=24)	56.1 (16.0)	50.6 (17.3)	-5.5 (-12.3, 1.3)	0.11		
Naïve: Memory helper T cells						
HIIT (n=18)	1.28 (0.81)	1.27 (0.72)	-0.02 (-0.15, 0.12)	0.81	-0.04 (-0.29, 0.20)	0.72
UC (n=24)	0.79 (0.49)	0.92 (0.58)	0.13 (-0.05, 0.30)	0.15		
Cytotoxic T cells^*^ (CD3+CD8+)						
HIIT (n=21)	23.3 (11.8)	23.1 (11.8)	-0.2 (-1.6, 1.2)	0.76	-0.4 (-4.1, 3.3)	0.82
UC (n=24)	33.0 (10.7)	31.8 (9.9)	-1.2 (-4.2, 1.8)	0.41		
LAMP-1 on activated cytotoxic T cells^*^ (CD107a+ in CD3+CD8+)						
HIIT (n=16)	7.0 (3.7)	6.1 (3.4)	-0.9 (-3.3, 1.5)	0.45	1.0 (-1.0, 3.0)	0.33
UC (n=24)	5.5 (2.7)	5.2 (2.2)	-0.3 (-1.5, 0.9)	0.62		
Activated (CD25/IL-2Rα+) cytotoxic T cells^*^ (CD25+ in CD3+CD8+)						
HIIT (n=17)	3.3 (2.8)	2.7 (1.9)	-0.6 (-1.6, 0.5)	0.26	-0.5 (-2.4, 1.4)	0.61
UC (n=23)	2.4 (1.9)	2.8 (3.5)	0.4 (-1.2, 2.0)	0.63		
Major NK-cell subset (CD3-CD16+CD56+)						
HIIT (n=19)	9.1 (4.0)	11.1 (3.7)	2.0 (0.4, 3.6)	**0.018**	2.0 (0.3, 3.7)	**0.024**
UC (n=22)	10.7 (6.0)	9.9 (4.8)	-0.8 (-2.2, 0.5)	0.21		
NK-associated subset (CD27+CD16+CD56+)						
HIIT (n=16)	3.4 (1.4)	2.4 (1.2)	-1.0 (-2.0, 0.0)	0.055	-0.3 (-1.2, 0.5)	0.41
UC (n=24)	3.8 (2.5)	2.7 (1.3)	-1.1 (-2.1, -0.1)	**0.040**		
PBMC-mediated cytotoxicity against K562 (†% cytotoxicity)						
HIIT (n=18)	15.4 (6.7)	17.7 (7.7)	2.4 (0.7, 4.0)	**0.008**	2.9 (0.3, 5.4)	**0.031**
UC (n=18)	17.4 (7.6)	16.8 (6.3)	-0.6 (-2.7, 1.5)	0.546		
Total B cells (CD19+)^*^						
HIIT (n=20)	21.2 (6.2)	20.0 (6.6)	-1.3 (-4.2, 1.7)	0.38	0.4 (-5.6, 6.3)	0.90
UC (n=24)	19.8 (7.3)	19.7 (11.7)	-0.1 (-5.5, 5.3)	0.97		
CD11c+ memory B cells (CD11c+CD19+)						
HIIT (n=14)	13.1 (4.0)	11.2 (4.3)	-1.8 (-5.4, 1.8)	0.29	1.3 (-2.4, 5.0)	0.48
UC (n=17)	12.2 (4.5)	9.7 (5.3)	-2.4 (-5.6, 0.7)	0.12		
IgM+ B cells (IgM+CD19+)						
HIIT (n=19)	6.6 (2.9)	6.4 (3.0)	-0.2 (-1.7, 1.3)	0.80	0.4 (-1.1, 2.0)	0.57
UC (n=24)	4.7 (2.4)	5.2 (2.0)	0.5 (-0.4, 1.4)	0.24		

* Log-transformation was used for normality and variance stabilization. T-test was used for within-group differences, and analysis of covariance was used for between-group differences adjusting for baseline values of the outcome and resistance exercise behavior. † % Cytotoxicity = (absorbance sample- Spontaneous LDH activity)/(Maximum LDH activity - spontaneous LDH activity) x 100. HIIT, high-intensity interval training; UC, usual care; IL-2, interleukin-2; LAMP-1, Lysosome-Associated Membrane Protein 1; NCAM, neural cell adhesion molecule. Denominators differ by row: values are % of PBMC for total T-, B-, and NK-cell populations; helper (CD3+CD4+) and cytotoxic (CD3+CD8+) T-cell subsets are proportions within the parent lymphocyte/CD3+ gate; and activation (CD25/IL-2Rα+), naïve (CD45RA+), and memory (CD45RO+) subsets are proportions of the respective CD4+ or CD8+ parent gate. The major NK-cell subset was defined as CD3−CD56+CD16+ (CD3− exclusion applied); the CD27+CD16+CD56+ NK-associated subset was measured in a separate tube without a CD3 channel, so CD3−/NKT exclusion could not be applied to that subset. † Cytotoxicity reflects PBMC-mediated cytotoxicity against K562 cells (LDH release) and cannot be normalized to a fixed NK-cell number. Per-outcome sample sizes vary owing to assay failure, insufficient sample volume, or removal of statistical outliers identified by studentized residuals.

Bold values indicate statistical significance (p < 0.05).

The change in cytokine levels from baseline to postintervention by randomized group are presented in **Table 4** and **Figure 1C**. Compared to UC group, plasma level of IL-4 showed a significant decrease in the HIIT group (adjusted between-group difference, -0.02 pg/ml; 95% CI, -0.03 to -0.01; *p* = 0.011). Similarly, plasma level of IL-12p70 was also significantly decreased in the HIIT group (adjusted between-group difference, -0.14 pg/ml; 95% CI, -0.23 to -0.06; *p* = 0.002). The proportion of other cytokines IL-2, IL-6, IL-8, IL-10, IL-13, IFN-γ, TNF-α or C-reactive protein did not show statistically significant within-group changes or between-group differences.

**Table 4:** Effects of high-intensity interval training on inflammatory cytokines in men with prostate cancer under active surveillance.

Variables	Baseline	Postintervention	Within-group mean change		Adjusted between-group difference	
	Mean (SD)	Mean (SD)	Mean (95% CI)	*P*	Mean (95% CI)	*P*
IL-2^*^ (pg/ml)						
HIIT (n=21)	0.52 (0.29)	0.40 (0.15)	-0.12 (-0.24, 0.01)	0.061	-0.13 (-0.29, 0.03)	0.10
UC (n=25)	0.89 (0.68)	0.85 (0.71)	-0.04 (-0.14, 0.05)	0.33		
IL-4 (pg/ml)						
HIIT (n=22)	0.06 (0.05)	0.05 (0.01)	-0.01 (-0.04, 0.01)	0.29	-0.02 (-0.03, -0.01)	**0.011**
UC (n=24)	0.06 (0.02)	0.06 (0.02)	0.00 (0.00, 0.01)	0.11		
IL-6 (pg/ml)						
HIIT (n=23)	1.12 (0.58)	0.98 (0.46)	-0.14 (-0.37, 0.09)	0.22	-0.10 (-0.33, 0.13)	0.38
UC (n=23)	0.96 (0.54)	0.97 (0.51)	0.00 (-0.13, 0.14)	0.95		
IL-8^*^ (pg/ml)						
HIIT (n=23)	2.25 (0.79)	2.44 (1.05)	0.19 (-0.15, 0.53)	0.26	0.09 (-0.43, 0.60)	0.74
UC (n=23)	2.10 (0.65)	2.20 (1.01)	0.11 (-0.28, 0.49)	0.57		
IL-10^*^ (pg/ml)						
HIIT (n=23)	0.46 (0.23)	0.39 (0.17)	-0.07 (-0.16, 0.01)	0.079	-0.06 (-0.13, 0.02)	0.13
UC (n=25)	0.41 (0.21)	0.41 (0.19)	0.00 (-0.04, 0.04)	0.96		
IL-12p70^*^ (pg/ml)						
HIIT (n=22)	0.30 (0.18)	0.25 (0.11)	-0.05 (-0.12, 0.02)	0.13	-0.14 (-0.23, -0.06)	**0.002**
UC (n=25)	0.49 (0.41)	0.53 (0.41)	0.04 (-0.02, 0.10)	0.21		
IL-13^*^ (pg/ml)						
HIIT (n=22)	2.01 (0.96)	1.79 (0.73)	-0.22 (-0.56, 0.11)	0.18	-0.41 (-0.97, 0.16)	0.15
UC (n=25)	3.32 (2.33)	3.37 (2.49)	0.05 (-0.34, 0.43)	0.80		
IFN-γ^*^ (pg/ml)						
HIIT (n=21)	6.58 (3.35)	6.72 (3.29)	0.14 (-0.96, 1.23)	0.80	-0.42 (-1.87, 1.03)	0.56
UC (n=21)	5.98 (2.88)	6.61 (3.49)	0.63 (-0.37, 1.63)	0.20		
TNF-α^*^ (pg/ml)						
HIIT (n=24)	0.97 (0.43)	0.98 (0.47)	0.01 (-0.07, 0.10)	0.74	0.01 (-0.10, 0.11)	0.89
UC (n=23)	0.87 (0.32)	0.88 (0.34)	0.01 (-0.03, 0.06)	0.55		
CRP (mg/L)						
HIIT (n=20)	2.86 (2.43)	2.25 (2.12)	-0.61 (-1.58, 0.36)	0.20	-0.45 (-1.44, 0.55)	0.37
UC (n=22)	2.10 (1.83)	2.07 (1.65)	-0.03 (-0.61, 0.55)	0.92		

* Log-transformation used for normality and variance stabilization. T-test was used for within-group differences, and analysis of covariance was used for between-group differences adjusting for baseline values of the outcome and resistance exercise behavior. IL, interleukin; IFN, interferon; TNF, tumor necrosis factor; CRP, c-reactive protein; HIIT, high-intensity interval training; UC, usual care. IL-4 and IL-12p70 concentrations clustered near the lower end of the assay working range and should be interpreted with caution; no values required exclusion or imputation. Estimates for log-transformed outcomes are reported on the log scale as indicated. Per-outcome sample sizes vary owing to assay failure, insufficient sample volume, or removal of statistical outliers identified by studentized residuals.

Bold values indicate statistical significance (p < 0.05).

In exploratory moderation models, none of the interaction terms reached statistical significance (all p ≥ 0.157; **Table 5**), and the confidence intervals were wide with very small model-specific samples; these underpowered, hypothesis-generating analyses carry no evidentiary weight. We therefore do not interpret the individual effect-size estimates (e.g., NK cytotoxicity or IL-12p70) as indicating a mechanistic association with the intervention effect on PSA.

**Table 5:** Moderation analysis of the effects of HIIT intervention on PSA and LNCaP outcomes by selected immune biomarkers.

Moderator	Interaction term (B [95% CI])	Standardized β	R²	*P*
PSA				
IL-4	-0.58 (-7.24, 6.07)	-0.125	0.064	0.867
IL-12p70	-2.02 (-9.63, 5.60)	-0.253	0.081	0.600
NK cell (%)	0.02 (-0.33, 0.37)	0.067	0.042	0.899
NK cytotoxicity	0.11 (-0.15, 0.37)	0.492	0.102	0.398
LNCaP				
IL-4	-0.20 (-0.90, 0.49)	-0.424	0.250	0.559
IL-12p70	-0.01 (-0.09, 0.08)	-0.052	0.126	0.899
NK cell (%)	0.05 (-0.02, 0.12)	0.165	0.209	0.157
NK cytotoxicity	0.04 (-0.02, 0.10)	0.243	0.246	0.168

PSA, prostate specific antigen; IL, interleukin; NK, natural killer.

## Discussion

To our knowledge, the ERASE trial is the first randomized controlled study to investigate the effects of supervised high-intensity interval training (HIIT) on a comprehensive panel of circulating immune cells and cytokines in men with localized prostate cancer on active surveillance. The main findings from this secondary analysis indicate that a 12-week HIIT program significantly increased the proportion of the circulating major NK-cell subset (CD3−CD56+CD16+) and enhanced PBMC-mediated cytotoxicity against K562 compared to UC. These changes were accompanied by significant reductions in the plasma concentrations of interleukin-4 (IL-4) and interleukin-12p70 (IL-12p70). Conversely, the intervention did not induce significant changes in major leukocyte populations such as T cells, B cells, or neutrophils, nor in other key inflammatory cytokines like IL-6 and TNF-α. While HIIT favorably modulated specific immune parameters, these changes did not significantly moderate the intervention’s effect on prostate cancer biomarkers in exploratory analyses. Collectively, these results provide novel evidence that HIIT can selectively modulate important components of the innate immune system in this specific clinical setting.

The observed increase in the major NK-cell subset and enhanced PBMC-mediated cytotoxicity aligns with extensive literature demonstrating that NK cells are the one of the most exercise-responsive immune cell populations. NK cells represent first line of innate immune defense against cancer cells, possessing natural killing ability without prior antigenic exposure. Exercise-induced NK cell mobilization occurs through catecholamine-driven mechanisms, particularly epinephrine release, which enhances both circulating NK cell numbers and their tumor infiltration capacity (15). In the HIIT group, PBMC-mediated cytotoxicity increased by an adjusted between-group difference of 2.9 percentage points (95% CI, 0.3 to 5.4) and a within-group change of 2.4 percentage points (from 15.4% to 17.7%); the latter corresponds to a relative within-group increase of approximately 15%, which should not be conflated with the between-group treatment effect. This magnitude is broadly consistent with previous exercise oncology studies reporting improvements in NK-cell function following structured exercise interventions (9, 16–18).

The significant decreases in IL-4 and IL-12p70 following HIIT, while small in absolute terms, suggest complex immune regulatory changes. IL-4 is a prototypical Th2 cytokine that promotes humoral immunity and can suppress cellular immune responses crucial for tumor surveillance (19). Regular aerobic or moderate‐intensity exercise has been shown to reduce circulating IL-4 levels in cancer patients, including those with solid tumors, indicating a downregulation of Th2‐mediated inflammation (20). For example, a randomized trial of aerobic training in cancer patients demonstrated significant decreases in IL-4, alongside improvements in cardiorespiratory fitness and reductions in other pro‐inflammatory factors such as TNF-α and Vascular Endothelial Growth Factor (VEGF). Similarly, exergaming and structured activity programs reduced Th2 cytokines (including IL-4) and regulatory cytokines such as IL-10 and transforming growth factor (TGF)-β, suggesting a partial rebalancing toward Th1 dominance (21). The reduction in IL-4 in our study may suggest the decline of Th2-dominated immunity, potentially favoring Th1 responses that are more effective for anti-tumor activity (22). On the other hand, the concurrent decrease in IL-12p70 (-0.14 pg/ml) is biologically ambiguous and should be interpreted with caution, particularly because both IL-4 and IL-12p70 clustered near the lower end of the assay working range, so these small absolute changes approach the assay resolution. IL-12 is central to Th1 polarization, NK activation, and cytotoxic T-cell support, so a reduction is not unequivocally favorable: although it may reflect a dampening of systemic/chronic inflammation, it could equally reflect a broader reduction in inflammatory signaling rather than a targeted beneficial effect, and we do not interpret it as evidence of enhanced anti-tumor immunity (23, 24). Taken together with the decrease in IL-4, these cytokine changes may indicate a shift in systemic inflammatory signaling, but their direction of benefit remains uncertain (25).

The immune system changes observed in ERASE demonstrate meaningful integration with other trial outcomes, particularly PSA kinetics and LNCaP cell growth suppression. The ERASE trial showed that HIIT significantly reduced PSA levels (-1.1 μg/L) and PSA velocity (-1.3 μg/L/year), alongside a 5.1% reduction in LNCaP cell growth (11). These biochemical improvements align with literature demonstrating that exercise-conditioned serum can suppress prostate cancer cell growth through multiple mechanisms.​ For example, studies using LNCaP cell lines have consistently shown that serum from exercised individuals inhibits cancer cell proliferation by 17-31% compared to serum collected in the resting state (26). This tumor-suppressive effect appears mediated by exercise-induced myokines, including oncostatin M, IL-6, IL-15, and decorin, which are secreted by skeletal muscle during contraction (27, 28). Furthermore, we examined exploratory moderation analyses for the interaction between immune markers and clinical outcomes; none of the interaction terms was statistically significant (all p ≥ 0.157), and given the wide confidence intervals and very small model-specific samples, these underpowered analyses carry no evidentiary weight. We therefore do not infer a mechanistic link between NK cytotoxicity (or any other immune marker) and the PSA or LNCaP outcomes, and present these results only as hypothesis-generating.

The immune system modifications observed in ERASE have several important clinical implications for men with prostate cancer undergoing active surveillance. The enhanced NK cell function represents a potentially crucial mechanism for improved immune surveillance of malignant cells (9). Given that NK cells can recognize and eliminate cancer cells without prior sensitization, the observed increases in the NK-cell proportion and PBMC-mediated cytotoxicity may be relevant to immune surveillance; however, these peripheral-blood changes do not by themselves establish improved tumor control during active surveillance (29). Additionally, the shift in cytokine profile away from IL-4 dominance, combined with the increase in NK-cell activity, suggests that HIIT may alter the circulating immune profile, although the tumor-specific relevance of these peripheral changes remains unestablished. This is particularly relevant in the active surveillance setting, where the goal is to maintain disease stability while avoiding unnecessary treatment-related toxicities. The co-occurrence of these circulating immune changes with the previously reported biochemical (reduced PSA kinetics) and ex vivo anti-proliferative (LNCaP cell growth) findings is consistent with exercise acting through multiple complementary pathways, a hypothesis that requires direct tumor-level confirmation. Finally, the specific HIIT protocol used in ERASE (85-95% of VO_2_peak for 2-minute intervals) might have been more effective for immune system activation, in comparison to other studies employing moderate-intensity continuous training (30). However, the high-intensity nature of the intervention likely contributed to the robust catecholamine responses necessary for optimal NK cell mobilization and cytokine modulation (31). That said, the dose-response relationship between exercise intensity and immune function is not yet fully established, and definitive clinical evidence is warranted.

It is important to emphasize that an increase in the circulating major NK-cell subset or in PBMC-mediated cytotoxicity against K562 may not, by itself, establish improved control of the prostate tumor. Exercise may raise the number or activity of circulating immune cells without necessarily improving their access to, or function within, the tumor microenvironment (32). Mechanisms of immune exclusion, including tumor and myeloid PD-L1 expression, which engages PD-1 on cytotoxic T and NK cells to suppress their activation and infiltration, can limit the tumor relevance of peripheral immune changes (33, 34). Although preclinical and translational work suggests that exercise can enhance intratumoral cytotoxic-cell infiltration and may modulate PD-L1 expression (15, 35–38), our study did not measure tumor-level readouts, and we therefore cannot infer effects on tumor-specific immunity.

Furthermore, exercise immunology in cancer is context-dependent, and not all peripheral immune shifts should be assumed to be favorable; indeed, most immune endpoints in our study were unchanged. A recent systematic review and meta-analysis found that exercise did not consistently alter tumor-specific immune-cell activity in breast cancer patients and survivors (39), underscoring that the relationship between exercise-induced peripheral immune changes and tumor-relevant immunity is not established as uniformly beneficial. We therefore interpret our selective findings cautiously and avoid generalizing them to anti-tumor immunity.

Our resting cytokine data also warrant a balanced treatment of IL-6. Because post-intervention samples were drawn at least 72 hours after the last session, our IL-6 values reflect the chronic, resting state rather than the acute, contraction-induced myokine spike; the absence of a between-group change in resting IL-6 should therefore not be read as an absence of an acute IL-6 response (40). Mechanistically, this distinction is important because repeated acute elevations of IL-6 could, via JAK/STAT3 signaling, upregulate PD-L1 on tumor and myeloid cells and thereby suppress cytotoxic T- and NK-cell activation and infiltration, potentially counteracting the proposed anti-tumor benefits of exercise (41). This double-edged potential suggests that future studies should characterize both acute and chronic IL-6 dynamics, distinguish classic from trans-signaling using the soluble IL-6 receptor (sIL-6R) (42), and evaluate whether combining exercise with immune-checkpoint inhibition mitigates any IL-6/PD-L1-mediated impairment of tumor immune infiltration (36).

The ERASE trial represents several important strengths in exercise immunology research. It is the first randomized controlled trial to comprehensively examine immune system responses to exercise specifically in men with prostate cancer undergoing active surveillance. The supervised HIIT intervention with excellent adherence (96%) ensures intervention fidelity, while the comprehensive immune phenotyping using flow cytometry and multiplex cytokine analysis provides robust mechanistic insights. The integration of immune markers with functional outcomes (NK cell cytotoxicity) and clinical endpoints (PSA, LNCaP cell growth) offers a translational perspective linking immune changes to potential clinical benefits. However, several limitations should be noted. The relatively small sample size (n=52) may have limited statistical power for detecting subtle immune changes and especially for the moderation analyses. The 12-week intervention period, while sufficient to demonstrate short-term training adaptations, may not capture longer-term immune system adaptations that could be more clinically relevant; the ≥72-hour post-exercise sampling window was designed to capture the resting/chronic state rather than the acute, contraction-induced myokine response. Also, the study population was predominantly White (89%) and relatively homogeneous in terms of cancer risk profile, potentially limiting generalizability to more diverse populations or higher-risk disease states. In addition, dietary intake and nutritional status were not monitored during the 12-week period, which could have confounded the cytokine and inflammatory outcomes. Statistically, we did not adjust for multiple comparisons; given the large number of comparisons (20+ endpoints), the individual findings remain exploratory and require confirmation in independent, adequately powered studies. Finally, the immune assessments were limited to circulating markers and did not include tissue-based immune infiltration studies, which would provide more direct evidence of anti-tumor immune responses. Building on these findings, future work should determine whether these immune adaptations track with concurrent changes in functional outcomes such as cardiorespiratory fitness and muscular strength. Complementary markers of IL-6 signaling, particularly the soluble IL-6 receptor (sIL-6R), should also be incorporated to distinguish classic from trans-signaling and better characterize the inflammatory versus myokine-mediated actions of exercise-induced IL-6.

Several phenotyping and assay limitations should also be acknowledged. Because the CD56+CD16+ gate was applied directly to CD3− cells, a CD16-independent CD3−CD56+ population (including the immunoregulatory CD56bright CD16−/dim subset) was not captured, so our results reflect the major CD16+ NK-cell subset rather than total NK cells. The CD27+CD16+CD56+ NK-associated subset was measured in a tube lacking a CD3 channel and without CD11b, so contamination by CD3+CD56+ (NKT-like) cells cannot be excluded and full NK maturation staging was not possible. Because CCR7 was not included in the panel, CD45RA+ cells cannot be defined as strictly naïve (terminally differentiated effector-memory cells re-express CD45RA), and memory/differentiation status was not assessed for CD8+ T cells owing to panel design. Finally, because the cytotoxicity assay used bulk PBMCs, the observed change cannot be normalized to a fixed NK-cell number and most likely reflects a combination of the higher NK-cell proportion and any change in per-cell function rather than intrinsic per-cell NK cytotoxicity; cryopreservation may also affect NK-cell viability and function, although paired pre- and post-intervention samples from both arms were thawed and analyzed together on the same day.

## Conclusion

These findings suggest that HIIT can modulate selected circulating innate immune parameters in men with prostate cancer on active surveillance. The clinical and tumor-specific relevance of these changes requires further investigation in larger, longer-term studies with tumor-level immune readouts.

## Data Availability

The datasets generated and analyzed during this study are not publicly available because of participant privacy and consent restrictions. De-identified data are available from the corresponding author on reasonable request.
